# Supplementary material for: Local’s attitude towards African elephant conservation in and around Chebra Churchura National Park, Ethiopia
Source: PLoS One. 2023 Oct 26;18(10):e0292641. doi: 10.1371/journal.pone.0292641 (PMC10602352; doi:10.1371/journal.pone.0292641)
Supplement: Attachment — Submitted filename: Response to Reviewers.docx [file pone.0292641.s002.docx]

**Dear Editor,**

We have corrected all editorial comments and suggestions and the questions raised by both reviewers are clarified. Specific comments for each reviewer are clarified below:

1. We have cheeked and confirmed that our manuscript meets PLOS ONE's style requirements, including those for file naming.

2. Financial disclosure:

Funded studies
Funded by Rufford Small Grant
Grant Number: ID:39147-2
Grant Awarded to the corresponding Author ADANE TSEGAYE TEGEGNE

"The funders had no role in study design, data collection and analysis, decision to publish, or preparation of the manuscript.

3. We declared that no authors have competing interests

Bewick et al. [5]) which has currently not yet been accepted for publication. Please remove this from your References and amend this to state in the body of your manuscript: (ie “Bewick et al. [Unpublished]”) as detailed online in our guide for authors
Please upload the completed Content Permission Form or other proof of granted permissions as an "Other" file with your submission.

4. We want to make a change to our Data Availability statement; all data are included in the manuscript. We have downloaded the excel data as “Supporting Information files” separately

5. You said that we have referenced (ie. Bewick et al. [5]) which has currently not yet been accepted for publication, but I did not use this reference in the text or in the reference list

6. We have removed Figure 1 from the manuscript.”

7. We have reviewed the reference list to ensure that it is complete and correct and confirmed it is complete and correct.

**Response to comments from reviewer 1**

1. Concerning the prepositions ‘in’ and ‘around’ in the title **Local People’s Attitude towards African elephant Conservation in and around Chebra Churchura National Park, Ethiopia.** Even though the data collection, interviews were conducted from 800 households distributed in 20 park adjacent villages totally out of the park boundary our intension includes investigating the local communities attitude if it is totally against elephant conservation whether it is inside the park or outside. We want to answer the question if it is it the elephants attack in the surrounding communities that shaped their attitude towards the elephant conservation. If that is the case they will have positive attitude towards the elephant conservation as long as the elephants do not come out of the park and attack them. On the other hand if their attitude is not attached to the elephants attack they will develop the same positive or negative attitudes irrespective of the elephants attack so that they will not be interested in implementing HEC mitigation measure. However, if using the preposition in do not sounds good and make the title explanatory we are willing to remove the preposition “in” from it.
2. We have cheeked and corrected the spelling of the households (written as hose holds) in the Abstract.
3. In the Introduction section, we added one additional paragraph that explain about the importance and significance of the research topic (HEC or HWC) by relating the topic with the relevant sustainable development goals.
4. We have corrected the spelling of livelihoods (mis-spelled as lively hoods) in the section given before the section Study Area:
5. In the section Questionnaire Design we have corrected the spelling of chi-square test (written as chi-square taste).
6. In Table 4, we have correct the spelling of crop teff, (mis-spelled as tefee),
7. We have given complete form of the acronyms HEC, CCNP and HWC at the very first places where the acronyms are used. Subsequently, we used as abbreviated throughout the manuscript.
8. In the section Implications for Conservation: we have corrected acronym CCNP in place of CCNNP.
9. We added a text that explains about the originality or novelty of the study just before the acknowledgment section.
10. Sampling technique was clarified

    **Response to Reviewer 2.**
1. Abstract must be short and crisp. We have removed a paragraph which was unnecessary details of results in abstract which seems a repetition when is read in result section. We removed 4 lines between line 14 and 19 which was described as “Most respondents (38%) reported firing warning gun by park scouts as an effective method of crop prevention followed by Chilly and bee hive fencing (19.7%) sound noise including the sound of barking dog and hammering materials made of metal (13.3%)	guarding	(11.3%)	fire smoking (9%) and smoking chilly and elephant dung (9%)”
2. Based on the reviewers comment we explained the result in the separate section “entitled” focus group discussion.
